# Obesity and Other Correlates of Physical Activity and Sedentary Behaviors among US High School Students

**DOI:** 10.1155/2013/276318

**Published:** 2013-03-31

**Authors:** Richard Lowry, Sarah M. Lee, Janet E. Fulton, Zewditu Demissie, Laura Kann

**Affiliations:** ^1^Division of Adolescent and School Health, National Center for HIV/AIDS, Viral Hepatitis, STD, and TB Prevention, Centers for Disease Control and Prevention, 4770 Buford Highway, NE (Mailstop K-33), Atlanta, GA 30341, USA; ^2^Division of Population Health, National Center for Chronic Disease Prevention and Health Promotion, Centers for Disease Control and Prevention, Atlanta, GA 30341, USA; ^3^Division of Nutrition, Physical Activity, and Obesity, National Center for Chronic Disease Prevention and Health Promotion, Centers for Disease Control and Prevention, Atlanta, GA 30341, USA

## Abstract

Understanding correlates of physical activity (PA) can help inform and improve programs that promote PA among youth. We analyzed data from the 2010 National Youth Physical Activity and Nutrition Study, a representative sample of US students in grades 9–12. Logistic regression was used to examine associations between PA correlates (obesity, physical education classes, sports team participation, attitude toward PA, adult support for PA, and environmental support for PA) and participation in daily PA (DPA), vigorous PA (VPA), muscle-strengthening activity (MSA), viewing television (TV), and using computers or video games (C/VG). A positive attitude toward PA and adult support for PA were both associated with increased PA and decreased sedentary behavior. However, among students who lived in neighborhoods that were not safe for PA, a positive attitude toward PA was not associated with increased DPA or decreased sedentary behavior and was less strongly associated with VPA and MSA. Efforts to increase PA among youth should promote a positive attitude toward PA among youth and encourage adult family members to support their efforts to be active. Policies that promote safe neighborhoods may work synergistically with a positive attitude toward PA to increase participation in PA and decrease sedentary behaviors.

## 1. Introduction

In the United States, approximately one out of three adolescents and two out of three adults are either overweight or obese [1, 2]. Regular physical activity can improve the health and quality of life of all Americans, including those who are overweight or obese [3]. For people who are inactive, even small increases in physical activity are associated with health benefits [3, 4]. Among adults, physical activity can lower the risk of early death, depression, coronary heart disease, stroke, high blood pressure, type 2 diabetes, and obesity [4]. Among children and adolescents, physical activity can reduce symptoms of depression and stress, improve cardiorespiratory and muscular fitness, improve bone health, and decrease levels of body fat [4]. Unfortunately, participation in physical activity declines dramatically during adolescence [5]. The physical activity objectives for Healthy People 2020 reflect the 2008 Physical Activity Guidelines for Americans which recommend that children and adolescents get at least 60 minutes (1 hour) of daily physical activity that consists mostly of moderate- to vigorous-intensity aerobic physical activity and that includes vigorous-intensity physical activities (at least 3 days per week), muscle-strengthening physical activities (at least 3 days per week), and bone-strengthening physical activities (at least 3 days per week) [3, 4]. The Healthy People 2020 physical activity objectives also address sedentary behaviors, by calling for an increase in the proportion of children and adolescents who do not exceed recommended limits for screen time, including viewing television no more than 2 hours a day and using a computer or playing computer games outside of school (for nonschool work) no more than 2 hours a day [4].

 Personal, social, and environmental factors all play a role in determining physical activity levels among youth [4, 5]. Physical activity researchers have identified some of the principal factors found to be positively associated with physical activity among adolescents which include parental education, male gender, participation in physical education classes and school sports, belief in ability to be active (self-efficacy), personal goals, enjoyment of physical activity, support of friends and family, and supportive environments (e.g., presence of sidewalks, access to neighborhood or school play areas, and access to recreational equipment) [4–9]. In addition, youth report neighborhood crime/danger as a barrier to physical activity [10] and youth who perceive their neighborhood as unsafe are less likely to be physically active [11–14]. One possible mechanism whereby neighborhood safety may affect participation in physical activity is by acting as an effect modifier and changing the effect of other physical activity correlates on participation in physical activity and sedentary behaviors. Understanding the perceived barriers and facilitators of physical activity is important to ensure the effectiveness of neighborhood, community, and school interventions, programs, policies, and practices to improve levels of physical activity among youth. 

This study extends the literature by examining the associations of obesity with physical activity and sedentary behaviors within the context of other known correlates and determinants of physical activity levels in youth, including attitude toward physical activity, adult support for physical activity, and environmental support for physical activity. In addition, since a lack of neighborhood safety has been shown to be a barrier to participation in physical activity, we examined whether perceived neighborhood safety modifies the associations between other physical activity correlates and participation in physical activity and sedentary behaviors.

## 2. Methods

### 2.1. Study Design

We analyzed data from the National Youth Physical Activity and Nutrition Study (NYPANS), a cross-sectional, school-based study conducted in 2010 by the Centers for Disease Control and Prevention (CDC) to collect information on physical activity and dietary behaviors and the determinants of those behaviors among adolescents. NYPANS used a three-stage cluster-sample design to obtain a nationally representative sample of students in grades 9 through 12 who attend public and private high schools in the 50 states and the District of Columbia. Students completed a self-administered questionnaire in their classrooms during a regular class period in the spring of 2010. The school response rate was 82%, the student response rate was 88%, and the overall response rate was 73%. Usable questionnaires were returned by 11,429 students. A weighting factor was applied to each student record to adjust for nonresponse and oversampling of African-American/black and Hispanic/Latino students. Student participation in the study was anonymous and voluntary, and the parental permission procedures utilized in each sampled school were in line with the local school policies. NYPANS was approved by the study contractor's (ICF Macro) institutional review board.

### 2.2. Measures

#### 2.2.1. Physical Activity and Sedentary Behaviors (Outcomes)

 Participation in moderate- to vigorous-intensity daily physical activity (DPA) was assessed by asking, “During the past 7 days, on how many days were you physically active for a total of at least 60 minutes per day? (Add up all the time you spent in any kind of physical activity that increased your heart rate and made you breathe hard some of the time.)” DPA was coded as 7 days versus <7 days. Vigorous physical activity (VPA) was assessed by asking, “On how many of the past 7 days did you exercise or participate in physical activity for at least 20 minutes that made you sweat and breathe hard, such as basketball, soccer, running, swimming laps, fast bicycling, fast dancing, or similar aerobic activities?” Regular participation in VPA was coded as ≥3 days versus <3 days. Muscle-strengthening activity (MSA) was assessed by asking, “On how many of the past 7 days did you do exercises to strengthen or tone your muscles, such as push-ups, sit-ups, or weight lighting?” Regular participation in MSA was coded as ≥3 days versus <3 days. Television (TV) viewing was assessed by asking, “On an average school day, how many hours do you watch TV?” Excessive TV viewing was coded as ≥3 hours versus <3 hours. Computer and video game (C/VG) use was assessed by asking, “On an average school day, how many hours do you play video or computer games or use a computer for something that is not school work? (Include activities such as Nintendo, Game Boy, PlayStation, Xbox, computer games, and the Internet.)” Excessive C/VG use was coded as ≥3 hours versus <3 hours. Although we could have analyzed these outcomes as continuous variables or ordinal variables with multiple levels (e.g., 0 days versus 1, 2, 3, 4, 5, 6, or 7 days of physical activity), we chose instead to dichotomize these physical activity and sedentary outcomes to be consistent with current national health objectives and federal guidelines [3, 4]. 

#### 2.2.2. Correlates of Physical Activity (Independent Variables)

 Demographic variables included sex, race/ethnicity (non-Hispanic white, non-Hispanic black, Hispanic, and other), and grade (9th, 10th, 11th, 12th). Body mass index (BMI) was calculated from measured height and weight. Before measurements were taken, students were asked to remove outer clothing (e.g., coats), purses, shoes, hats, and any removable hair accessories and to remove personal items from their pockets. The data collectors measured height to the nearest inch using a weighted measuring tape attached to the wall. Students placed their backs and heels against the wall, and the data collectors then placed a measuring triangle on the student's head to form a right angle with the wall. The height measurement was taken from the lower edge of the triangle. Data collectors measured students' weight to the nearest pound using a Tanita electronic scale placed on an uncarpeted floor. The scale was zero balanced before each student was weighed. Based on reference data from growth charts produced by CDC, students with a BMI ≥95th percentile for sex and age were considered to be obese. Participation in physical education (PE) classes was assessed by asking, “In an average week when you are in school, on how many days do you go to physical education (PE) classes?” Daily PE classes were coded as 5 days versus <5 days. Participation in sports teams was assessed by asking, “During the past 12 months, on how many sports teams did you play? (Include teams run by your school or community groups.)” Sports team participation was coded as ≥1 team versus 0 teams. Attitude toward physical activity was assessed by a series of 5 questions utilizing a 5-point Likert scale, adult support for physical activity was assessed by a series of 4 questions utilizing a 5-point frequency scale, and environmental support for physical activity was assessed by a series of 3 questions utilizing a 5-point Likert scale (Table 1). We combined survey items to create scales for “attitude toward physical activity” (5 items, Cronbach alpha = 0.88) and “parental support for physical activity” (4 items, Cronbach alpha = 0.80). Because the Cronbach alphas for these scales were high (i.e., ≥0.8) we included these survey items as scales in logistic regression models. The “environmental support for physical activity” scale had a low Cronbach alpha (<0.6), indicating that the 3 “environmental support for physical activity” questions did not function together well as a scale; therefore, we chose to enter these as separate independent variables in logistic regression models.

### 2.3. Data Analyses

Data were weighted to provide national estimates and analyzed using SUDAAN version 10.0.1 (Research Triangle Institute, Research Triangle Park, NC). First, we calculated prevalence estimates with 95% confidence intervals (CIs) for physical activity and sedentary behaviors, demographic characteristics, BMI category, and other correlates of physical activity (participation in PE classes and sports teams; sports equipment at home; playgrounds, parks, or gyms close to home; neighborhood safe for physical activity; personal attitude toward physical activity; and adult support for physical activity). Bivariate analyses were conducted using Chi-square testing to identify significant (*P* < 0.05) differences in the prevalence of physical activity and sedentary behaviors by sex, race/ethnicity, grade, and BMI category. Next, logistic regression was used to estimate both unadjusted odds ratios (ORs) and adjusted odds ratios (AORs) for associations of obesity and other correlates of physical activity with participation in physical activity and sedentary behaviors. AORs simultaneously controlled for sex, race/ethnicity, grade, obesity, and other correlates of physical activity. All odds ratios were considered statistically significant if *P* < 0.05. Finally, we tested for interactions between neighborhood safety and other correlates of physical activity to determine whether living in a neighborhood that is safe for physical activity modified the associations between other correlates of physical activity and participation in physical activity and sedentary behaviors. 

## 3. Results

### 3.1. Demographic Characteristics

The weighted distribution of the demographic characteristics of students in the NYPANS sample was 49.4% female, 50.6% male; 57.7% non-Hispanic white, 14.9% non-Hispanic black, 18.9% Hispanic, and 8.5% of other race/ethnicity; 27.8% in 9th grade, 25.9% in 10th grade, 23.8% in 11th grade, and 22.5% in 12th grade.

### 3.2. Physical Activity and Sedentary Behaviors

Among all students, 15.1% participated in at least 60 minutes of DPA, 69.7% participated in VPA for ≥20 minutes on ≥3 days per week, and 50.7% participated in MSA on ≥3 days per week (Table 2). Approximately one out of four students viewed TV ≥3 hours a day (28.3%) and used C/VG ≥3 hours a day (23.5%). 

### 3.3. Correlates of Physical Activity

 Based on measured BMI, 19.0% of students were obese, 17.8% were overweight, 60.7% were normal weight, and 2.5% were underweight (Table 2). Among all students, 36.3% participated in daily PE classes and 61.0% played on ≥1 sports teams. Most students either agreed or strongly agreed that they had access to sports equipment at home (70.7%), playground, parks, or gyms close to home (68.4%), and neighborhoods that were safe for physical activity (73.5%). Also, most students agreed or strongly agreed with each of the 5 positive statements about physical activity, indicating a generally positive attitude toward physical activity. Finally, most students received some support for physical activity from adults in their household. However, less than half (48.5%) of students reported that adults in their household engaged in physical activity with them during a typical week. 

### 3.4. Physical Activity and Sedentary Behaviors by Demographics and BMI Category

 In general, participation in physical activity was less common among female students, black and Hispanic students, students in higher grade levels, and obese students, while excessive TV viewing was more common among female students, black and Hispanic students, and obese students (Table 3). Excessive C/VG use was more common among male students and black students.

### 3.5. Associations with Physical Activity and Sedentary Behaviors

Table 4 shows the prevalence of the outcome behavior among students without and with the correlate present. For example, among students who are not obese, 16.2% engaged in DPA, while only 10.6% of obese students engaged in DPA. In unadjusted logistic regression models (Model 1), obesity was associated with decreased odds of participation in DPA (OR = 0.61) and MSA (OR = 0.77) and increased odds of excessive TV viewing (OR = 1.47) (Table 4). The other correlates of physical activity were positively associated (OR > 1) with participation in DPA, VPA, and MSA and, except for participation in daily PE classes, were negatively associated (OR < 1) with excessive TV viewing and C/VG use.

 In adjusted logistic regression models (Model 2), which simultaneously controlled for sex, race/ethnicity, grade, obesity, and other correlates of physical activity, obesity was independently associated with decreased odds of participation in DPA (AOR = 0.76), increased odds of participation in VPA (AOR = 1.23), and excessive TV viewing (AOR = 1.29) (Table 4). Participation in daily PE classes and sports teams was each associated with increased odds of DPA, VPA, and MSA. Participation in sports teams, but not PE, was associated with decreased odds of excessive TV viewing and C/VG use. Having sports equipment at home was positively associated with DPA and VPA and negatively associated with excessive TV viewing. Having playgrounds, parks, or gyms close to home was not significantly associated with participation in any physical activity or sedentary behaviors, and living in a neighborhood that was safe for physical activity was only associated with decreased odds (AOR = 0.73) of participation in DPA. A positive attitude toward physical activity (high scale scores) was strongly associated with increased physical activity and decreased sedentary behaviors. Adult support for physical activity (high scale scores) was also associated with increased physical activity and decreased odds of excessive C/VG use.

### 3.6. Effect Modification by Neighborhood Safety

 To determine whether living in a safe neighborhood acted as an effect modifier, we tested for interactions between living in a safe neighborhood and each of the other correlates of physical activity. A highly significant (*P* < 0.001) interaction was found between neighborhood safety and attitude toward physical activity for each physical activity and sedentary behavior, except for excessive C/VG use (*P* = 0.13) (Table 5). 

 Finally, because of the significant interactions between neighborhood safety and attitude toward physical activity, we examined the associations between attitude toward physical activity and participation in physical activity and sedentary behaviors, stratified by neighborhood safety (Table 5). Among students who lived in neighborhoods that were safe for physical activity, a positive attitude toward physical activity was associated with increased odds of participation in DPA, VPA, and MSA and decreased odds of excessive TV viewing and C/VG use. However, among students who lived in neighborhoods that were not safe for physical activity, a positive attitude toward physical activity was not associated with increased DPA or with decreased TV and C/VG use and was less strongly associated with participation in VPA and MSA. 

## 4. Discussion

 This study builds upon existing research that has reported on youth physical activity behaviors and their correlates and provides additional insight into some physical activity correlates not adequately studied in previous research [6, 15]. Consistent with previous research, our results indicate that females, older adolescents, and black and Hispanic youth are less likely to participate in physical activity compared to males, younger adolescents, and white youth, respectively [6, 15]. The high prevalence of sedentary behaviors (i.e., TV and C/VG use) among black and Hispanic students in our study also is consistent with previous research which has documented higher levels of electronic media use and inactivity among black and Hispanic youth, compared to white youth [6, 15, 16]. It is important that health promotion efforts seek not only to increase physical activity, but also to decrease time spent in sedentary behavior, because these are independent rather than mutually exclusive behaviors, and there are subgroups of youth who are both highly active and highly sedentary [17]. 

 In our study, as expected, obese youth were less likely to participate in DPA and were more likely to watch an excessive amount of TV. However, after controlling for demographic characteristics and other correlates of physical activity, obese students were more likely than nonobese students to participate in VPA. It is possible that this unexpected finding is the result of obese students being more likely than nonobese students to perceive moderate-intensity physical activity such as brisk walking (perhaps done for weight control purposes) as vigorous-intensity physical activity. 

 A unique contribution of our study is that we looked at participation in school PE classes as a potential determinant of DPA, VPA, and MSA among students. This has rarely been done, particularly with a nationally representative sample of high school students. Our results indicate that participation in daily PE is positively associated with DPA, VPA, and MSA, an encouraging finding. Daily PE is recommended by the CDC, the National Association for Sport and Physical Education (NASPE), and is identified as a key priority in the U.S. National Physical Activity Plan [18–20]. Unfortunately, only 4% of elementary schools, 8% of middle schools, and 2% of high schools, nationally, provide daily PE for students [21]. Schools can promote physical activity by offering students PE daily or at least regularly throughout the school year. In high school, it is particularly important to provide students with daily opportunities for physical activity, because participation in DPA decreases with age [5, 6]. Similar to daily PE classes, sports team participation was also associated with increased DPA, VPA, and MSA. However, unlike participation in PE classes, participation in sports teams was associated with decreased participation in sedentary behaviors. This is not surprising, since students who choose to participate in sports teams may prefer to spend their time in active rather than sedentary pursuits and also might have less time to use TV and C/VG because of their attendance at team practices and games.

 All three environmental supports for physical activity (sports equipment at home, playgrounds/parks close to home, and neighborhood safe for physical activity) were associated with increased physical activity and decreased sedentary behaviors in unadjusted models. However, in adjusted models controlling for demographic characteristics, obesity, and the other correlates of physical activity, only having sports equipment at home was associated with increased participation in DPA and VPA and decreased TV viewing. This association is likely to be bidirectional; having sports equipment at home may encourage students to be more physically active and less sedentary, and students who prefer physical activity over sedentary pursuits may be more likely to have sports equipment at home. The fact that neighborhood safety was associated with decreased participation in DPA in the adjusted model was an unexpected and possibly misleading finding. Because of the strong statistical interaction (i.e., effect modification) between neighborhood safety and attitude toward physical activity, it is difficult to accurately interpret the effect of neighborhood safety as an independent variable in the adjusted model when attitude toward physical activity is also in the model. Since the association between attitude toward physical activity and participation in physical activity and sedentary behaviors depends upon whether or not the neighborhood was safe for physical activity, we chose to stratify the analysis by neighborhood safety and examine the effect of attitude toward physical activity separately among students who lived in safe or unsafe neighborhoods (Table 5). 

 Consistent with previous research on correlates and determinants of physical activity among youth, students who had a positive attitude toward physical activity, who had the support of adults in their household to be physically active, and who played in sports teams were more likely to be physically active and less likely to be sedentary [5, 6, 15]. The fact that neighborhood safety interacts strongly with a student's attitude toward physical activity, thereby changing the association between students' attitude toward physical activity and their participation in physical activity and sedentary behaviors, is a particularly interesting finding from our study. In the stratified analysis, among students who lived in neighborhoods that they perceived to be safe for physical activity, a positive attitude toward physical activity was associated with increased physical activity and decreased sedentary behaviors. However, among students who lived in neighborhoods that they perceived to be unsafe for physical activity, a positive attitude toward physical activity did not remain associated with increased DPA or decreased TV and C/VG use and was less strongly associated with participation in VPA and MSA. Violence presents a significant barrier to active lifestyles and healthy living in communities, but the provision of safe, attractive, and accessible parks and other green spaces offers a promising strategy for promoting physical activity and reducing sedentary behavior among youth [22–24]. A recent study found that greening of urban vacant land was associated with significant reductions in violence, and also with resident reports of less stress and more exercise [23]. Without safe places to play near home, children may spend more time being sedentary indoors [24].

 The findings in this report are subject to several limitations and caveats. First, these data apply only to youths who attend school and therefore are not representative of all persons in this age group. Nationwide, in 2009, of persons aged 16-17 years, approximately 4% were not enrolled in a high school program and had not completed high school [25]. Second, due to the lack of objective measures of physical activity, the extent of underreporting or overreporting of self-reported behaviors could not be determined, although the survey questions demonstrate good test-retest reliability [26]. For example, it is possible that our finding of greater participation in VPA among obese youth compared to nonobese youth may reflect a misperception of the amount or intensity of physical activity among less physically fit obese youth rather than an actual increase in participation in vigorous intensity physical activity. Third, the data are cross-sectional, and therefore causality and directionality of associations cannot be determined. Finally, NYPANS data cannot isolate the effects of race/ethnicity from the effects of other factors on the prevalence of physical activity and sedentary behaviors. Although participation in physical activity and sedentary behaviors varied among racial and ethnic subgroups, additional research is needed to assess the effects of education, socioeconomic status, and cultural factors on the prevalence of these behaviors and to help intensify physical activity promotion efforts in the communities where inactivity is most heavily concentrated.

## 5. Conclusions

 A breadth of research exists that has shown how perception of neighborhood safety influences participation in physical activity [11–14, 22], and a number of studies also have examined attitude toward physical activity and its association with participation in physical activity [5, 6]. Our study looked at both of these potential determinants and their interaction. The findings suggest that the beneficial effects of a positive attitude toward physical activity, which is often a major determinant of increased participation in physical activity and possibly decreased participation in sedentary behaviors, can be diminished or even negated by living in a neighborhood that is perceived as being unsafe for physical activity. This is an important finding for public health practitioners, schools, communities, and parents. All of these groups can be influential in promoting physical activity participation among adolescents. However, without broad and effective support for improved neighborhood safety, it may be difficult to achieve substantial progress toward increasing physical activity and reducing sedentary behaviors among youth.

 Increasing physical activity and decreasing sedentary behaviors among youth will require that communities work together with schools and families to provide safe, attractive, and accessible places close to home, where they can be active [27]. Our findings suggest that interventions designed to increase physical activity among youth by promoting a positive attitude toward physical activity may be most effective if measures are taken to provide youth with opportunities to be physically active in venues they perceive as safe. Further research is needed to identify those factors which most influence perceptions of neighborhood safety among youth. Communities can help by providing neighborhood parks that are safe for physical activity with safe walking trails or paths and safe play areas and by encouraging community organizations to offer supervised physical activity programs for youth which would likely be perceived by youth as safer than physical activity without adult supervision. Schools can work with community organizations to create and implement safe routes to school programs, which encourage more children to safely walk and bike to school, and to offer before- and after-school physical activity programs and events. Schools should also offer recess and in-class physical activity breaks for younger students, intramural sports opportunities that offer a choice of activities for students of all skill levels, and quality daily PE classes for all students. Our findings also suggest a strong association between adult support for physical activity and greater participation in physical activity and less sedentary behavior among youth. Consequently, families and adult caregivers should encourage youth to be physically active instead of watching television or playing video games. Caregivers can encourage youth to be active not only by helping them to participate in team or individual sports, but also by being physically active with them by building physical activity into family time through noncompetitive activities such as walking, bicycling, hiking, jogging, and swimming. 

## Figures and Tables

**Table 1:** Psychometric properties of scales used to measure correlates of physical activity (PA), including personal attitude, adult support, and environmental support among US high school students.

Scale name no. of items (score range)	Mean (SE)	Cronbach alpha (scale)	Concept questionnaire items
Attitude toward PA 5 items^a^ (5–25)	20.7 (0.1)	0.88	When I am physically active:
			I enjoy it.
			I find it fun.
			It gives me energy.
			My body feels good.
			It gives me a strong feeling of success.

Adult support for PA 4 items^b^ (4–20)	9.8 (0.1)	0.80	During a typical week how often does an adult in your household:
			Encourage you to do physical activities or play sports?
			Do a physical activity or play sports with you?
			Provide transportation to a place where you can do physical activities or play sports?
			Watch you participate in physical activities or sports?

Environmental support for PA 3 items^a^ (3–15)	11.6 (0.1)	0.58	How much do you agree or disagree that:
			At home there are enough pieces of sports equipment (such as balls, bicycles, and skates) to use for physical activity.
			There are playgrounds, parks, or gyms close to my home that are easy for me to get to.
			It is safe to be physically active by myself in my neighborhood.

^a^Measured on a 5-point Likert scale: 1 = strongly disagree; 2 = disagree; 3 = neither agree nor disagree; 4 = agree; 5 = strongly agree.

^b^Measured on a 5-point frequency scale: 1 = never; 2 = 1-2 times/week; 3 = 3-4 times/week; 4 = 5-6 times/week; 5 = daily.

**Table 2:** Unweighted frequency and weighted prevalence of physical activity (PA) and sedentary behaviors, body mass index (BMI) categories, and other PA correlates among US high school students.

Category	*n*	%	(95% CI)
PA behaviors			
Daily PA (DPA) (≥60 min/day, 7 days/wk)	1628	15.1	(13.8, 16.6)
Vigorous PA (VPA) (≥20 min/day, ≥3 days/wk)	7710	69.7	(67.6, 71.7)
Muscle strengthening activity (MSA) (≥3 days/wk)	5669	50.7	(48.3, 53.0)
Sedentary behaviors			
Television (TV) (≥3 hrs/day)	3734	28.3	(25.7, 31.0)
Computer/video games (C/VG) (≥3 hrs/day)	2778	23.5	(21.8, 25.2)
BMI category			
Obese (≥95th percentile)	1922	19.0	(17.3, 21.0)
Overweight (≥85th to <95th percentile)	1845	17.8	(16.8, 18.9)
Normal weight (≥5th to <85th percentile)	6002	60.7	(58.6, 62.8)
Underweight (<5th percentile)	238	2.5	( 2.0, 3.0)
Behavioral correlates of PA			
Daily physical education (PE) classes (5 days/wk)	3833	36.3	(30.4, 42.6)
Sports team participation (≥1 team, past 12 months)	6699	61.0	(58.3, 63.6)
Environmental support for PA (agree/strongly agree)			
Sports equipment at home to use for PA	7637	70.7	(68.2, 73.1)
Playgrounds, parks, or gyms close to home	7624	68.4	(65.0, 71.6)
Neighborhood safe for PA by myself	7746	73.5	(70.5, 76.4)
Attitude toward PA (agree/strongly agree)			
I enjoy it	9271	82.5	(81.0, 83.8)
I find it fun	8754	78.1	(76.6, 79.6)
It gives me energy	8441	75.6	(74.1, 77.0)
My body feels good	8573	78.2	(76.5, 79.8)
It gives me a strong feeling of success	8589	77.6	(76.4, 78.8)
Adult support for PA (≥1 time/wk)			
Encourage you to do PA or play sports?	8223	73.9	(72.6, 75.1)
Do PA or play sports with you?	5436	48.5	(47.5, 49.6)
Provide transportation to PA or sports you do?	7445	67.8	(66.1, 69.5)
Watch you do PA or sports?	6699	61.8	(59.4, 64.0)

BMI: body mass index = weight (kg)/height (m)^2 ^(based on measured height and weight, using age- and sex-specific percentiles from growth charts developed by Centers for Disease Control and Prevention).

**Table 3:** Prevalence of participation in daily physical activity (DPA), vigorous physical activity (VPA), muscle-strengthening activity (MSA), television (TV) viewing, and computer or video game (C/VG) use, by demographic and BMI category among US high school students.

Categorical variable	DPA	VPA	MSA	TV	C/VG
	% (95% CI)	% (95% CI)	% (95% CI)	% (95% CI)	% (95% CI)
Sex					
Male	21.7 (19.3, 24.4)	78.1 (76.1, 80.0)	64.4 (60.8, 67.9)	26.4 (24.0, 29.0)	27.6 (25.4, 30.0)
Female	8.4 (7.3, 9.5)	61.2 (58.1, 64.2)	36.6 (34.1, 39.1)	30.2 (26.9, 33.8)	19.2 (17.4, 21.3)
Chi Sq, 1 df (*P* value)	(0.0000)	(0.0000)	(0.0000)	(0.0193)	(0.0000)

Race/ethnicity					
White	17.0 (15.6, 18.4)	72.3 (70.0, 74.5)	51.3 (48.4, 54.3)	20.6 (18.3, 23.2)	20.6 (18.7, 22.6)
Black	14.1 (12.0, 16.4)	65.1 (61.7, 68.4)	47.5 (44.3, 50.6)	52.7 (50.0, 55.5)	31.5 (28.0, 35.3)
Hispanic	11.9 (8.9, 15.7)	66.8 (62.4, 70.9)	52.2 (48.2, 56.2)	33.7 (31.6, 35.9)	23.4 (19.9, 27.3)
Other	12.4 (8.7, 17.5)	67.5 (60.8, 73.6)	48.4 (42.6, 54.3)	24.8 (18.3, 32.7)	29.5 (25.7, 33.5)
Chi Sq, 3 df (*P* value)	(0.0051)	(0.0001)	(0.1664)	(0.0000)	(0.0000)

Grade					
9th grade	18.0 (15.5, 20.8)	74.5 (69.8, 78.7)	55.6 (50.5, 60.5)	28.7 (25.2, 32.5)	23.4 (20.5, 26.5)
10th grade	15.4 (13.7, 17.4)	72.5 (69.7, 75.1)	51.1 (48.0, 54.2)	30.4 (26.7, 34.4)	24.4 (21.9, 27.2)
11th grade	13.7 (11.8, 15.9)	67.5 (64.9, 70.1)	48.9 (44.4, 53.4)	24.7 (21.5, 28.3)	23.2 (20.2, 26.5)
12th grade	12.7 (10.9, 14.8)	63.0 (59.9, 66.0)	45.8 (43.0, 48.6)	28.9 (25.2, 33.0)	22.8 (20.9, 24.8)
Chi Sq, 3 df (*P* value)	(0.0169)	(0.0000)	(0.0024)	(0.0274)	(0.3173)

BMI category					
Underweight/normal (<85th percentile)	16.2 (14.4, 18.2)	70.5 (68.2, 72.8)	52.3 (49.5, 55.0)	26.0 (23.2, 29.0)	23.6 (21.7, 25.5)
Overweight (≥85th to <95th percentile)	16.3 (13.3, 19.8)	70.0 (66.4, 73.4)	51.0 (46.6, 55.4)	29.6 (26.1, 33.4)	22.1 (19.8, 24.7)
Obese (≥95th percentile)	10.6 (8.2, 13.6)	67.8 (64.8, 70.7)	45.5 (42.1, 49.0)	35.1 (31.4, 38.9)	25.2 (21.7, 29.0)
Chi Sq, 2 df (*P* value)	(0.0002)	(0.1851)	(0.0012)	(0.0001)	(0.1470)

DPA: daily physical activity (≥60 min/day, 7 days/wk); VPA: vigorous physical activity (≥20 min/day, ≥3 days/wk); MSA: muscle strengthening activity (≥3 days/wk); TV: television (≥3 hrs/day); C/VG: computer or video games (≥3 hrs/day); BMI: body mass index = weight (kg)/height (m)^2 ^(based on measured height and weight, using age- and sex-specific percentiles from growth charts developed by Centers for Disease Control and Prevention).

**Table 4:** Prevalence, unadjusted (Model 1), and adjusted (Model 2) associations between physical activity (PA) correlates and PA and sedentary behaviors among US high school students.

PA correlates (independent variables)	Correlate not present (ref)	Correlate present	Model 1	Model 2
	% (95% CI)	% (95% CI)	OR	AOR
	Daily PA: ≥60 min/day, 7 days/week			

Obese (BMI ≥ 95th percentile)	16.2 (14.5, 18.1)	10.6 (8.2, 13.6)	0.61***	0.76*
Behavioral determinants of PA				
Daily PE classes	12.4 (10.9, 14.1)	19.7 (17.2, 22.5)	1.73***	1.40**
Sports team participation	7.9 (6.7, 9.3)	19.8 (18.0, 21.6)	2.88***	1.74***
Environmental support for PA				
Sports equipment at home	9.1 (7.6, 10.9)	17.5 (16.1, 19.1)	2.11***	1.24*
Playgrounds/parks close to home	12.8 (10.8, 15.0)	16.1 (14.4, 18.0)	1.31**	1.10
Neighborhood safe for PA	12.0 (10.1, 14.2)	16.2 (14.7, 17.9)	1.42***	0.73**
Attitude toward PA scale (score: 5–25)	—	—	1.12***	1.05**
Adult support for PA scale (score: 4–20)	—	—	1.14***	1.09***

	Vigorous PA: ≥20 min/day, ≥3 days/week			

Obese (BMI ≥ 95th percentile)	70.4 (68.1, 72.6)	67.8 (64.8, 70.7)	0.89	1.23**
Behavioral determinants of PA				
Daily PE classes	62.3 (60.1, 64.4)	82.8 (79.2, 85.8)	2.91***	2.80***
Sports team participation	53.4 (49.5, 57.4)	80.0 (78.4, 81.5)	3.49***	1.92***
Environmental support for PA				
Sports equipment at home	57.7 (54.4, 60.9)	74.7 (72.6, 76.7)	2.17***	1.20*
Playgrounds/parks close to home	64.4 (61.3, 67.3)	72.2 (69.9, 74.4)	1.44***	1.12
Neighborhood safe for PA	61.8 (59.0, 64.5)	72.6 (70.3, 74.8)	1.64***	0.94
Attitude toward PA scale (score: 5–25)	—	—	1.13***	1.08***
Adult support for PA scale (score: 4–20)	—	—	1.18***	1.12***

	Muscle-strengthening PA: ≥3 days/week			

Obese (BMI ≥ 95th percentile)	52.0 (49.4, 54.6)	45.5 (42.1, 49.0)	0.77***	0.96
Behavioral determinants of PA				
Daily PE classes	42.0 (39.9, 44.1)	65.9 (60.4, 70.9)	2.67***	2.57***
Sports team participation	36.0 (33.1, 38.9)	60.0 (57.5, 62.5)	2.68***	1.53***
Environmental support for PA				
Sports equipment at home	40.7 (37.4, 44.1)	54.8 (52.2, 57.3)	1.76***	1.05
Playgrounds/parks close to home	46.3 (43.5, 49.1)	52.7 (50.0, 55.3)	1.29***	0.98
Neighborhood safe for PA	43.9 (40.7, 47.1)	53.1 (50.2, 56.0)	1.45***	0.84
Attitude toward PA scale (score: 5–25)	—	—	1.13***	1.08***
Adult support for PA scale (score: 4–20)	—	—	1.14***	1.10***

	Television: ≥3 hours/day			

Obese (BMI ≥ 95th percentile)	26.8 (24.1, 29.7)	35.1 (31.4, 38.9)	1.47***	1.29*
Behavioral determinants of PA				
Daily PE classes	28.6 (26.0, 31.3)	27.8 (23.9, 32.0)	0.96	1.08
Sports team participation	34.7 (32.2, 37.3)	24.2 (21.3, 27.3)	0.60***	0.73***
Environmental support for PA				
Sports equipment at home	36.3 (33.2, 39.5)	24.8 (22.2, 27.5)	0.58***	0.79**
Playgrounds/parks close to home	30.5 (27.7, 33.4)	27.0 (24.1, 30.2)	0.84*	0.93
Neighborhood safe for PA	33.7 (30.9, 36.7)	26.2 (23.3, 29.3)	0.70***	1.05
Attitude toward PA scale (score: 5–25)	—	—	0.95***	0.96***
Adult support for PA scale (score: 4–20)	—	—	0.96***	0.99

	Computer/video games: ≥3 hours/day			

Obese (BMI ≥ 95th percentile)	23.2 (21.5, 25.0)	25.2 (21.7, 29.0)	1.11	1.05
Behavioral determinants of PA				
Daily PE classes	23.8 (21.9, 25.9)	22.9 (20.6, 25.4)	0.95	0.96
Sports team participation	30.0 (27.7, 32.5)	19.2 (17.5, 21.0)	0.55***	0.67***
Environmental support for PA				
Sports equipment at home	29.8 (26.8, 33.0)	20.8 (19.0, 22.6)	0.62***	0.82
Playgrounds/parks close to home	25.4 (22.8, 28.1)	22.5 (20.9, 24.1)	0.85*	1.03
Neighborhood safe for PA	26.9 (24.6, 29.4)	22.1 (20.3, 24.0)	0.77***	1.03
Attitude toward PA scale (score: 5–25)	—	—	0.93***	0.95***
Adult support for PA scale (score: 4–20)	—	—	0.93***	0.96*

OR: odds ratio, unadjusted; AOR: adjusted odds ratio, adjusted for sex, race/ethnicity, grade, and PA correlates. BMI: body mass index = weight (kg)/height (m)^2 ^(based on measured height and weight, using age- and sex-specific percentiles from growth charts developed by Centers for Disease Control and Prevention).

*P value < 0.05; **P value < 0.01; ***P value < 0.001.

**Table 5:** Interactions between neighborhood safety and attitude toward physical activity (PA) and associations between PA attitude and participation in PA and sedentary behaviors, by neighborhood safety among US high school students.

	DPA	VPA	MSA	TV	C/VG
Interactions: neighborhood safety × attitude toward PA					
Wald F, 1 df	22.8	14.4	12.7	15.8	2.3
(*P* value)	(0.0000)	(0.0004)	(0.0007)	(0.0002)	(0.1323)

	DPA	VPA	MSA	TV	C/VG
	AOR	AOR	AOR	AOR	AOR

Associations: attitude toward PA, by neighborhood safety					
Total population	1.05**	1.08***	1.08***	0.96***	0.95***
Neighborhood safe for PA	1.15***	1.11***	1.11***	0.94***	0.94***
Neighborhood not safe for PA	0.97	1.04**	1.04**	1.00	0.97

DPA: daily physical activity (≥60 min/day, 7 days/wk); VPA: vigorous physical activity (≥20 min/day, ≥3 days/wk); MSA: muscle strengthening activities (≥3 days/wk); TV: television (≥3 hrs/day); C/VG: computer or video games (≥3 hrs/day); AOR: adjusted odds ratio, adjusted for sex, race/ethnicity, grade, obesity, daily PE classes, sports team participation, sports equipment at home, playgrounds, parks, or gyms close to home, adult support for PA.

*P value < 0.05; **P value < 0.01; ***P value < 0.001.
